# Astragalus Polysaccharide Attenuates Cisplatin-Induced Acute Kidney Injury by Suppressing Oxidative Damage and Mitochondrial Dysfunction

**DOI:** 10.1155/2020/2851349

**Published:** 2020-01-03

**Authors:** Qing Ma, Yao Xu, Lumin Tang, Xiaoqian Yang, Zhejun Chen, Yuehan Wei, Xinghua Shao, Xiaoguang Shao, Zhixiang Xin, Biao Cai, Qi Wang, Shan Mou

**Affiliations:** ^1^Molecular Cell Laboratory for Kidney Disease, Department of Nephrology, Ren Ji Hospital, School of Medicine, Shanghai Jiao Tong University, 160 Pujian Road, Pudong New District, Shanghai, China; ^2^Department of Urology, Ren Ji Hospital, School of Medicine, Shanghai Jiao Tong University, 160 Pujian Road, Pudong New District, Shanghai, China

## Abstract

Cisplatin is a widely used chemotherapeutic drug in the treatment of various solid tumors. However, the cisplatin-induced acute kidney injury remains a disturbing complication, which still lacks effective prevention. Cisplatin-induced oxidative damage and mitochondrial dysfunction are anticipated to be crucial in the occurrence of kidney injury. Astragalus polysaccharide (APS) has been reported to possess multiple biological activities including anti-inflammatory, antioxidant, and mitochondria protection. In this study, we investigated the potentially protective effect of APS against cisplatin-induced kidney injury both in vivo and in vitro. We found that APS pretreatment attenuated the cisplatin-induced renal dysfunction and histopathological damage in mice; in addition, it also protected the viability of HK-2 cells upon cisplatin exposure. APS attenuated the cisplatin-induced oxidative damage by reducing reactive oxygen species (ROS) generation and recovering the activities of total superoxide dismutase and glutathione peroxidase in mice kidney. In addition, electron microscope analysis indicated that cisplatin induced extensive mitochondrial vacuolization in mice kidney. However, APS administration reversed these mitochondrial morphology changes. In HK-2 cells, APS reduced the cisplatin-induced mitochondrial and intracellular ROS generation. Furthermore, APS protected the normal morphology of mitochondria, blocked the cisplatin-induced mitochondrial permeability transition pore opening, and reduced the cytochrome *c* leakage. Subsequently, APS reduced the cisplatin-induced apoptosis in mice renal and HK-2 cells. In conclusion, our data suggested that APS pretreatment might prevent cisplatin-induced kidney injury through attenuating oxidative damage, protecting mitochondria, and ameliorating mitochondrial-mediated apoptosis.

## 1. Introduction

Cisplatin is a chemotherapeutic drug which is widely used in a variety of solid tumors [1]. Despite its effectiveness, the side effects including ototoxicity, hepatotoxicity, cardiac toxicity, and particularly cisplatin-induced acute kidney injury (AKI) are disturbing. About 30% of patients who received high-dose cisplatin suffered renal dysfunction, and the proportion was reported over 70% in pediatric patients [2]. Many studies have therefore made efforts to understand the potential mechanism of cisplatin-induced kidney injury, which may be helpful in exploring effective prevention [3–10].

The mechanism of cisplatin-induced nephrotoxicity is complex and involves many factors, including mitochondrial dysfunction, oxidative damage, and activation of apoptosis in renal tubular epithelial cells [6–10]. It is suggested that cisplatin can accumulate in mitochondria and cause mitochondrial damage, leading to reactive oxygen species (ROS) enrichment and kidney tubular cell death [6, 7]. In addition, cisplatin may cause mitochondrial fragmentation and membrane leakage, in which condition, apoptogenic proteins such as cytochrome *c* are leaked from the mitochondria into the cytoplasm and activates apoptosis in kidney tubular epithelial cells [8–10]. Therefore, pharmacological protection of mitochondria and inhibition of ROS stress are potential strategies to alleviate cisplatin-induced kidney injury.

Astragalus membranaceus is a kind of traditional Chinese medicine and has been widely used in therapy for a variety of diseases, including kidney lesions. Astragalus membranaceus has a complex chemical profile. Its major active constituents include Astragalus saponins, flavonoids, and polysaccharides [11]. Our previous works have demonstrated that Astragaloside IV (an active ingredient in Astragalus saponins) had potentially protective effects in obstructive nephropathy and cisplatin-induced nephrotoxicity [12, 13]. Astragalus polysaccharide (APS) is composed of glucans and heteropolysaccharide. Previous studies have indicated that APS might possess anti-inflammatory, antioxidant, and mitochondria protection properties, which could ameliorate inflammatory response, oxidative injury, and mitochondrial dysfunction in many different pathological conditions [14–18]. Furthermore, studies have suggested that APS may attenuate mitochondrial injury caused by ROS, possessing antiaging activity [19]. APS also could restore the morphologic changes induced by oxidative stress [20]. In a mouse Parkinson disease model, APS was reported to provide a protective effect on neurons by maintaining the mitochondrial structure and transmembrane potential [21]. In addition, APS could impede mitochondrial dysfunction and inhibit apoptosis in mesenchymal stem cells induced by iron overload [22].

Based on the main mechanism of cisplatin-induced kidney injury, combined with the protective effects of APS on oxidative damage and mitochondrial dysfunction, it is anticipated that APS holds potentially therapeutic effect for cisplatin nephropathy. Here, we investigated whether APS attenuated cisplatin-induced AKI through alleviating cisplatin-mediated oxidative damage, mitochondrial dysfunction, and renal tubular epithelial cell apoptosis.

## 2. Materials and Methods

### 2.1. Animal Model of Cisplatin-Induced Acute Kidney Injury and Drug Treatment

All animal experiments are in accordance with the appropriate institutional guidelines for animal research, and animal experiments are approved by Shanghai Jiao Tong University School of Medicine (approval no. SYXK2016-0009). 50 mg APS powder was added to 100 ml normal saline to generate APS solution. 8- to 10-week-old male C57BL/6 mice were randomly assigned to 3 groups: control group, *n* = 5: mice were intraperitoneally injected with normal saline; cisplatin-treated group, *n* = 5: mice were intraperitoneally injected with 30 mg/kg cisplatin (Hospira, Australia, Pty Ltd); and cisplatin- and APS-treated group, *n* = 5: mice were intraperitoneally injected with 30 mg/kg APS (MedChem Express) once a day for 10 consecutive days and injected with cisplatin at day 7. Mice were sacrificed after drug injection 72 h via pentobarbital sodium, and their blood and kidney samples were collected for further experiments. The serum creatinine level was determined using an automatic analyzer.

### 2.2. Human Kidney Tubular Cell Culture and the Determination of Cell Viability

HK-2 cells were purchased from American Type Culture Collection (ATCC, Manassas, VA) and cultured in DMEM/F12 (Gibco, America) containing 10% FBS at 37°C with 5% CO_2_. The cells were treated with four different drug strategies: the control group was treated with DMSO; the APS group was treated with APS (100 *μ*g/mL, MedChem Express) for 24 h; the cisplatin group was treated with cisplatin (20 *μ*mol/L, Hospira, Australia, Pty Ltd) for 24 h; and the APS and cisplatin group was pretreated with APS (100 *μ*g/mL, MedChem Express) for 24 h and then stimulated with cisplatin for 24 h. Cell viability was measured using a cell counting kit-8 (MedChem Express) and assessed by using a microplate reader (Thermo Fisher Scientific, Waltham, MA). Each experiment was repeated in triplicate.

### 2.3. Histological Analyses and Tubular Injury Score

Mice kidney tissues were fixed in 4% paraformaldehyde (PFA) for 24 h, embedded in paraffin wax, and cut into 5 *μ*m sections. Tissue sections were stained with hematoxylin and eosin (H&E) and periodic acid-Schiff (PAS), respectively, and then were examined under a light microscope. The tubular injury score was calculated based on the percentage of tubules displaying epithelial necrosis, cast formation, lumen dilatation, or the loss of brush border: 0, no damage; 1, 1–10% damage; 2, 11–25% damage; 3, 26–45% damage; 4, 46–75% damage; and 5, 76–100% damage [23]. 5 fields per section at ×200 magnifications were randomly selected and evaluated by a pathologist (Shao XH) in a blind manner.

### 2.4. Immunohistochemical Analyses

Paraffin tissue sections were used for immunohistochemical staining [24]. Briefly, these sections were deparaffinized with xylene and graded series of ethanol and then were treated with 10 mM citrate buffer (pH 6.0) for 30 min for antigen retrieval. The sections were then treated with 3% hydrogen peroxide to quench endogenous peroxidase activity. Sections were blocked with goat serum for 1 hour and then incubated with primary antibodies (rabbit anti-3-nitrotyrosine (3-NT) antibody, 1 : 1000, Abcam; and rabbit anti-kidney injury molecule-1 (KIM-1) antibody, 1 : 1000, Abcam) overnight at 4°C. Then, the sections were incubated with horseradish peroxidase- (HRP-) conjugated goat anti-rabbit IgG secondary antibody at room temperature for 1 h. Then, 3,30-diaminobenzidine (DAB) was used for the colorimetric immune reaction. Sections were counterstained with 0.1% hematoxylin. The expression of KIM-1 and 3-NT in tissue sections was evaluated under a light microscope.

### 2.5. ELISA

The activities of total superoxide dismutase (T-SOD) and glutathione peroxidase (GSH-Px) in mice kidney tissues were measured using commercial enzyme-linked immunosorbent assay (ELISA) kits (Beyotime, China), according to the protocols provided by the manufacturer.

KIM-1 level in serum and kidney was determined using a commercial ELISA kit (Liankebio, China), as specified by the instructions of the manufacturer.

### 2.6. Electron Microscopy

Mice renal tissue blocks (1 mm^3^) were fixed with glutaraldehyde (2.5%) at 4°C overnight. The tissues then underwent a series of chemical treatments (1% osmium tetroxide, distilled water, graded ethanol solutions, and propylene oxide) and were embedded in epoxy resin. After being sliced into ultrathin sections, tissues were stained with 4% uranyl acetate and 0.5% lead citrate. The mitochondrial morphology was observed and recorded using a transmission electron microscopy (Philips Tecnai 10).

### 2.7. TUNEL Assays

The apoptosis in mice renal tissue sections was determined using a deoxynucleotidyl transferase dUTP nick end-labeling (TUNEL) assay kit (Beyotime, China). Briefly, sections were deparaffinized and rehydrated with proteinase K; then, the sections were incubated with a TUNEL reaction buffer for 1 h. TUNEL-positive cells were counted in 5 fields per section at ×200 magnifications by a pathologist (Shao XH) in a blind manner.

TUNEL staining was also used to evaluate the antiapoptotic effect of APS on cisplatin-induced cell apoptosis. Briefly, HK-2 cells were fixed with 4% paraformaldehyde and permeabilized with 0.1% Triton X-100 and then were incubated with TUNEL reaction solution for 1 h at 37°C in darkness. At last, cells were treated with DAPI solution for 15 min. Cells were imaged randomly at ×200 magnifications using a fluorescence microscope. The TUNEL-positive cells ratio was defined as the number of TUNEL-positive cells divided by the total number of cells in a microscopy field at ×200 magnifications. TUNEL-positive cells ratio was calculated in 6 randomly selected fields.

### 2.8. Measurement of the Intracellular and Mitochondrial ROS

Predominant ROS in mitochondria was determined using MitoSOX Red mitochondrial superoxide indicator (Yeasen, Shanghai, China). Briefly, HK-2 cells were treated with strategies as previously described; cells were incubated with 5 *μ*M MitoSOX reagent working solution at 37°C for 15 min. Cells were then treated with DAPI solution for 15 min and imaged randomly at ×200 magnification using a fluorescence microscope. Mitochondrial ROS generation was shown as the fluorescence intensity of the red color. MitoSOX-positive cell ratio was calculated in 6 randomly selected fields per sample, and each experiment was repeated in triplicate.

Intracellular accumulation of ROS was evaluated using an oxidation-sensitive DCFH-DA kit (Sigma, the United States). Briefly, HK-2 cells were treated with strategies as previously described and then incubated with DCFH-DA (10 *μ*mol/L) in dark for 1 h. The cells were washed with DMEM/F12 culture medium and collected in PBS. The fluorescence of cells was measured by flow cytometer (Beckman, the United States), using 488 nm excitation and 525 nm emission wavelengths. Each experiment was repeated in triplicate.

### 2.9. Evaluation of Mitochondrial Morphology

Mitochondria were labeled with MitoTracker® Red CMXRos probe (Solarbio, China). Briefly, HK-2 cells were treated with strategies as previously described and incubated with MitoTracker® Red CMXRos working solution (100 nmol/L) in dark for 30 min. The cells were then washed with DMEM/F12 medium. Mitochondria were randomly imaged in 6 fields per sample at ×400 magnifications using a fluorescence microscope. The mitochondrial morphology was determined using ImageJ software as previously reported [25]: category 1 (elongated tubulin-like structure), category 2 (large round or short tubular), and category 3 (punctate). Each experiment was repeated in triplicate.

### 2.10. Measurement of Mitochondrial Permeability

Mitochondrial permeability transition pore (mPTP) opening in HK-2 cells was measured using the mPTP assay kit (Yeasen, Shanghai, China), according to the protocols provided by the manufacturer. Calcein AM is a dye that can selectively go through the mitochondrial membrane and produce green fluorescence. Calcein AM is released from the mitochondria when the mPTP opens, and the intensity of mitochondrial fluorescence reflects the degree of mPTP opening. The fluorescence intensity of mitochondria was determined by flow cytometer (Beckman, the United States), using 488 nm excitation and 525 nm emission wavelengths.

### 2.11. Western Blot Analyses

Total cytoplasmic protein was prepared by using cytoplasmic and mitochondrial protein extraction kit (Sangon Biotech). Briefly, the cells were harvested with 0.25% trypsin and then were centrifuged for 5 min at 3000 rpm at 4°C. The cells were then washed with PBS (pH 7.4) and centrifuged for 5 min at 3000 rpm at 4°C; the cells were suspended in cytoplasmic protein extraction buffer and placed on ice for 15 min and centrifuged for 10 min at 3000 rpm and 4°C, the resulting supernatant was centrifuged for 30 min at 12000 rpm and 4°C, and the resulting supernatant was the cytoplasmic protein. Protein concentrations were assessed using the BCA Protein Assay Kit. Equal amounts of protein were subjected to 12% SDS-PAGE gels and transferred to polyvinylidene difluoride membranes and blocked with 5% skim milk TBST (Tris-buffered saline Tween-20) buffer for 1 h. The membranes were incubated with anti-cytochrome *c* antibodies (1 : 1000, Abcam) and anti-GAPDH (1 : 1000, Abcam) overnight at 4°C; the membranes were then labeled with appropriate secondary antibodies for 1 h at room temperature and visualized by a CCD system (Tanon 2500R, Shanghai, China).

### 2.12. Statistical Analyses

All results are expressed as means ± SEM. Differences between groups were compared with one-way ANOVA followed by post hoc test. A *P* value <0.05 was considered statistically significant.

## 3. Results

### 3.1. APS Pretreatment Attenuates Cisplatin-Induced Acute Kidney Injury in Mice

**Figure 1:**
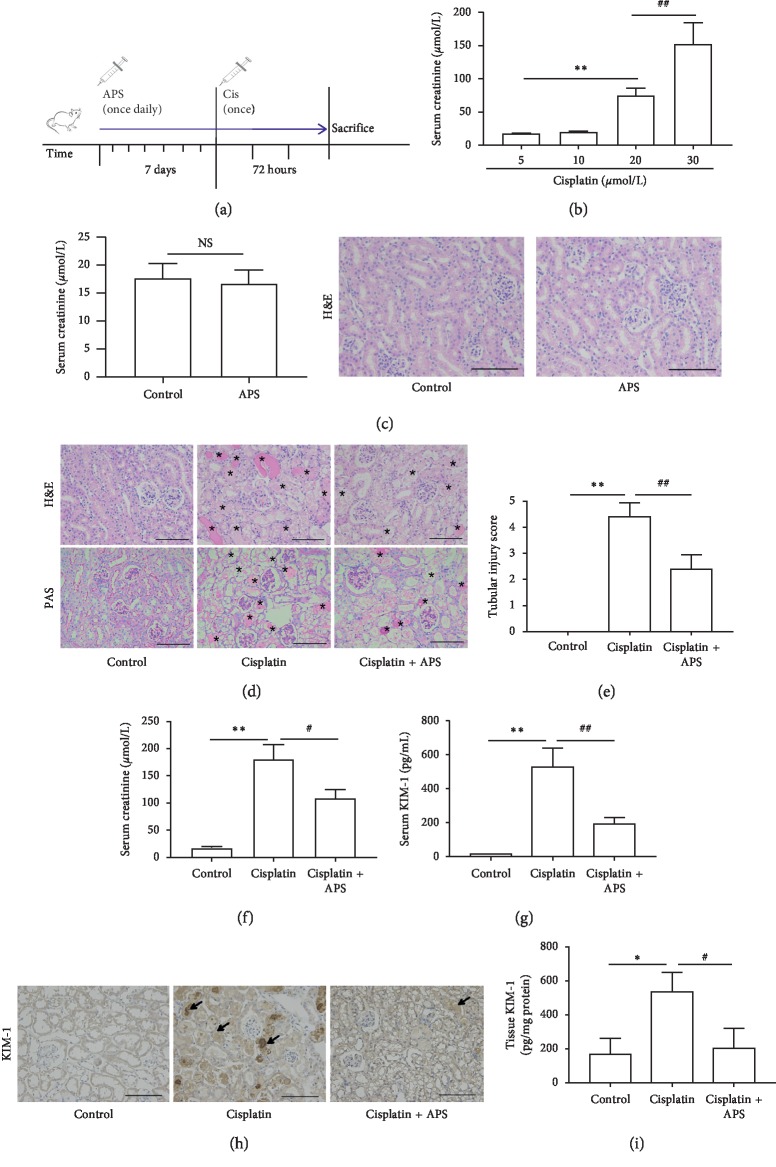
APS pretreatment attenuates cisplatin-induced acute kidney injury in mice. (a) Schematic diagram of mice treated with APS and cisplatin. (b) Mice were treated with different concentrations of cisplatin. Values are means ± SEM (*n* = 5). ^*∗∗*^*P* < 0.01, cisplatin 20 mg/kg group versus cisplatin 5 mg/kg group; ^##^*P* < 0.01, cisplatin 30 mg/kg group versus cisplatin 20 mg/kg group. (c) The serum creatinine level and renal histology of mice treated with APS (30 mg/kg) for 72 h (original magnification ×200). (d) Representative images of H&E staining and PAS staining of mice kidney tissue (original magnification ×200; bar = 250 *μ*m; ^*∗*^damaged tubules). (e) The tubular injury score is presented as means ± SEM in 5 random fields in each tissue. (f) Mice serum creatinine level. (g) Mice serum KIM-1 level. (h) Immunohistochemical staining of KIM-1 in mice kidney tissue (original magnification ×200; bar = 250 *μ*m). (i) ELISA analysis of KIM-1 protein in mice kidney tissue. Data are means ± SEM (*n* = 5). ^*∗*^*P* < 0.05, ^*∗∗*^*P* < 0.01 versus the control group; ^#^*P* < 0.05, ^##^*P* < 0.01 versus the cisplatin alone-treated group.

To investigate the effect of APS on cisplatin-induced AKI, we established the cisplatin-induced AKI animal model. The procedure of mice experiment and drug treatment is shown in Figure 1(a). We found that the serum creatinine level was elevated significantly and stably when the concentration of cisplatin was 30 mg/kg (Figure 1(b)), so we determined 30 mg/kg as the appropriate concentration of cisplatin in the following mice experiments. In addition, we found that APS did not affect the renal function (Figure 1(c)), indicating that APS could be safe in treatment.

Histological analyses showed that kidneys from the control mice had normal architecture, but those from cisplatin alone-treated mice displayed severe renal tubular injury, including tubular necrosis, tubular dilatation, cast formation, lumen dilatation, and loss of brush border. However, the renal tubular damage was significantly attenuated with APS pretreatment (Figure 1(d)), displaying lower tubular injury score in comparison with cisplatin alone-treated mice (Figure 1(e)). In addition, serum creatinine and serum KIM-1 levels in the APS pretreatment mice were significantly lower than those in the cisplatin alone-treated mice (Figures 1(f)–1(g)). Moreover, immunohistochemical staining and ELISA analysis both demonstrated that KIM-1 in renal tissue was markedly reduced in the APS pretreatment mice (Figures 1(h) and 1(i)).

### 3.2. APS Protects HK-2 Cells from Cisplatin Exposure

**Figure 2:**
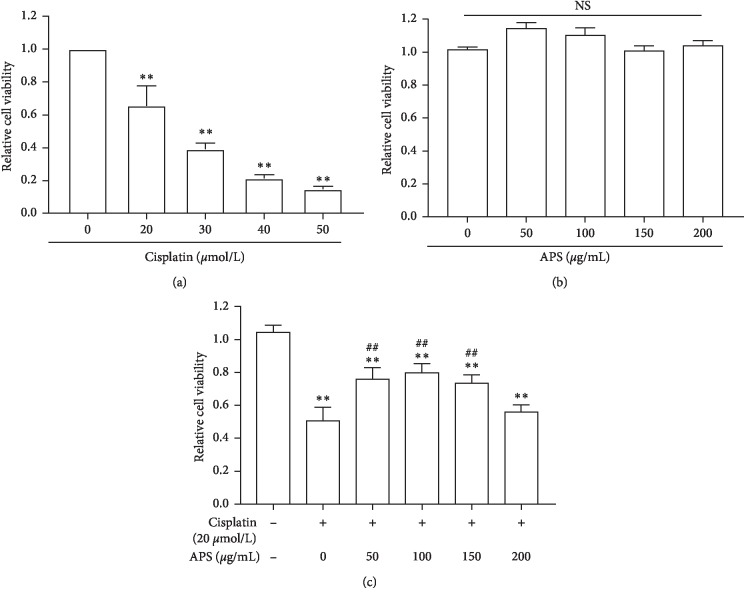
APS pretreatment protects HK-2 cells from cisplatin exposure. (a) HK-2 cell viability was measured by CCK8. Cells were incubated with different concentrations of cisplatin for 24 h from 0 (control) to 50 *μ*mol/L. (b) Cell viability of HK-2 cells treated with APS at different concentrations for 24 h from 0 (control) to 200 *μ*g/mL. (c) Cell viability of HK-2 cells treated with APS and cisplatin. HK-2 cells were pretreated with APS at different concentrations for 24 h and then were treated with 20 *μ*mol/L cisplatin for 24 h. Values are means ± SEM (*n* = 3). ^*∗∗*^*P* < 0.01, versus control; ^##^*P* < 0.01, versus the cisplatin alone-treated group.

Because cisplatin mainly affects the renal proximal tubular epithelial cells, we next explored the effect of APS on cisplatin-induced cytotoxicity using HK-2 cells. We firstly incubated cells with cisplatin at different concentrations (20, 30, 40, 60, 80, and 100 *μ*mol/L) for 24 h and measured the cell viability; we found that cisplatin-induced cytotoxicity was in a dose-dependent manner (Figure 2(a)). According to our results, we chose 20 *μ*mol/L in the following cell experiments, which induced about 40% cell death. We next determined the effect of APS on HK-2 cell proliferation, and we found that various doses of APS (50, 100, 150, and 200 *μ*g/mL) all had no cytotoxic effect (Figure 2(b)). Finally, we explored whether APS could protect the vitality of HK-2 cells upon cisplatin exposure and if so, the optimal dose at which this would happen. HK-2 cells were pretreated with APS at different concentrations from 0 to 200 *μ*g/mL for 24 h and then were treated with 20 *μ*mol/L cisplatin for 24 h. Results showed that 100 *μ*g/mL APS could protect cisplatin-induced HK-2 cell injury with maximal efficacy (Figure 2(c)). Therefore, we used 100 *μ*g/mL APS in the subsequent cell experiments.

### 3.3. APS Ameliorates Cisplatin-Induced Apoptosis in Mice Kidney and HK-2 Cells

**Figure 3:**
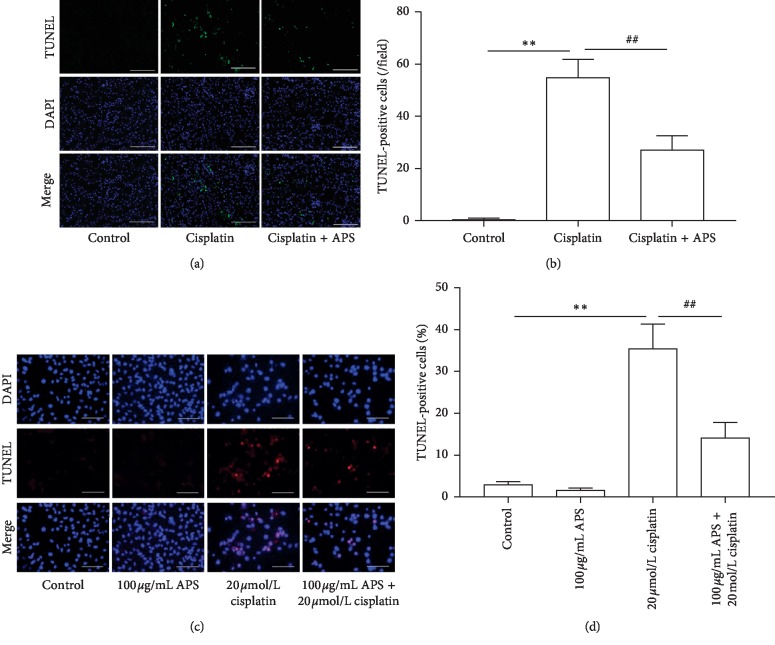
APS ameliorates cisplatin-induced apoptosis in vivo and in vitro. (a) TUNEL staining of mice kidney tissues (original magnification ×200; bar = 250 *μ*m). (b) Quantitation of TUNEL-positive cells in 5 random fields from each kidney. (c) TUNEL staining of HK-2 cells with different treatments. (d) Quantification of TUNEL-positive cell percentage in 6 random fields per sample. Original magnification ×200; bar = 250 *μ*m. Data are means ± SEM (*n* = 5). ^*∗∗*^*P* < 0.01 versus the control group; ^##^*P* < 0.01 versus the cisplatin alone-treated group.

Activation of apoptosis in renal tubular epithelial cells has been demonstrated in cisplatin-induced AKI [2]. Therefore, we explored whether APS could ameliorate cisplatin-induced apoptosis in mice kidney and HK-2 cells using TUNEL staining. Consistent with previous studies [24, 26], cisplatin induced significant cell apoptosis in both mice kidney and HK-2 cells, characterized by increased number of TUNEL-positive cells (Figures 3(a) and 3(c)). Interestingly, our results indicated that APS elicited an obviously protective effect by ameliorating the apoptosis, which was evidenced by a significantly reduced number of TUNEL-positive cells in mice kidney (Figures 3(a) and 3(b)) and HK-2 cells (Figures 3(c) and 3(d)).

### 3.4. APS Attenuates Cisplatin-Induced Oxidative Damage in Mice Kidney and HK-2 Cells

**Figure 4:**
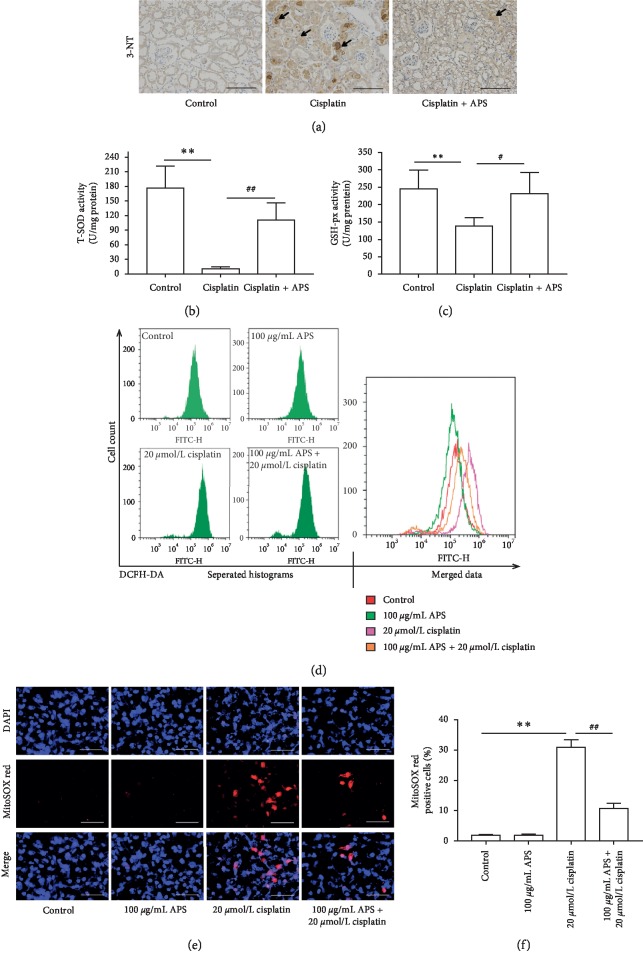
APS attenuates cisplatin-induced oxidative stress. (a) Immunohistochemical staining of 3-NT expression in mice kidney tissues (original magnification ×200; bar = 250 *μ*m). (b) T-SOD activity and (c) GSH-Px activity in mice kidney tissues. Data are means ± SEM (*n* = 5). ^*∗∗*^*P* < 0.01 versus the control group; ^#^*P* < 0.05, ^##^*P* < 0.01 versus the cisplatin alone-treated group. (d) APS reduced the intracellular ROS generation in HK-2 cells induced by cisplatin treatment. Separated and merged histograms of the DCFH-DA-stained HK-2 cells by flow cytometry. (e) APS significantly reduced the mitochondrial superoxide accumulation in HK-2 cells induced by cisplatin treatment. MitoSOX™ Red staining of HK-2 cells with different treatments (original magnification ×200; bar = 250 *μ*m). (f) Quantification of MitoSOX Red-positive cells in 6 random fields. Data are means ± SEM (*n* = 6). ^*∗∗*^*P* < 0.01 versus the control group; ^##^*P* < 0.01 versus the cisplatin alone-treated group.

Oxidative damage is one of the important pathogeneses by which cisplatin triggers kidney tissue injury [27]. In mice kidney tissues, cisplatin significantly increased oxidative stress marker 3-NT expression (Figure 4(a)) and inhibited antioxidant enzyme activities, including T-SOD and GSH-Px (Figures 4(b) and 4(c)), compared with the control group. Interestingly, these changes were markedly attenuated by APS administration. In addition, using an oxidation-sensitive DCFH-DA probe, we found that APS also reduced the intracellular ROS generation in HK-2 cells induced by cisplatin (Figure 4(d)).

Mitochondrial-dependent ROS response is the possible underlying mechanism of cisplatin-induced ROS generation [28]. Therefore, we evaluated the mitochondrial superoxide level using MitoSOX™ Red probe in HK-2 cells. As shown in Figure 4(e), cisplatin markedly stimulated the mitochondrial superoxide accumulation, leading to increased ratio of MitoSOX Red-positive cells. And APS had no effect on ROS generation. However, APS could significantly reduce the cisplatin-induced mitochondrial ROS accumulation (Figure 4(f)).

### 3.5. APS Attenuates the Cisplatin-Induced Mitochondrial Dysfunction

**Figure 5:**
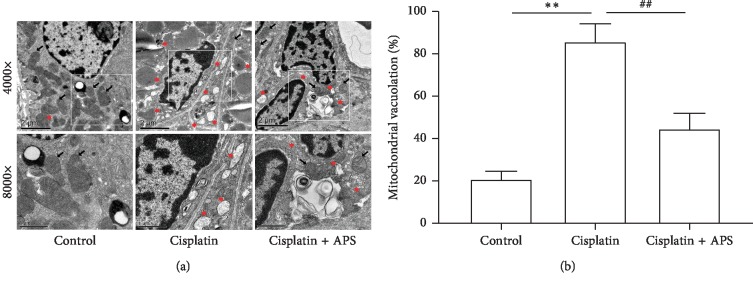
APS ameliorates the cisplatin-triggered mitochondrial damage in mice. (a) Transmission electron microscopy images of the mitochondrial morphology changes of mice renal (black arrow indicates normal mitochondria; red asterisk indicates damaged mitochondria; bar = 2 *μ*m and bar = 1 *μ*m). (b) Quantitative analysis of mitochondrial vacuolation. Data are means ± SEM (*n* = 3). ^*∗∗*^*P* < 0.01 versus the control group; ^##^*P* < 0.01 versus the cisplatin alone-treated group.

**Figure 6:**
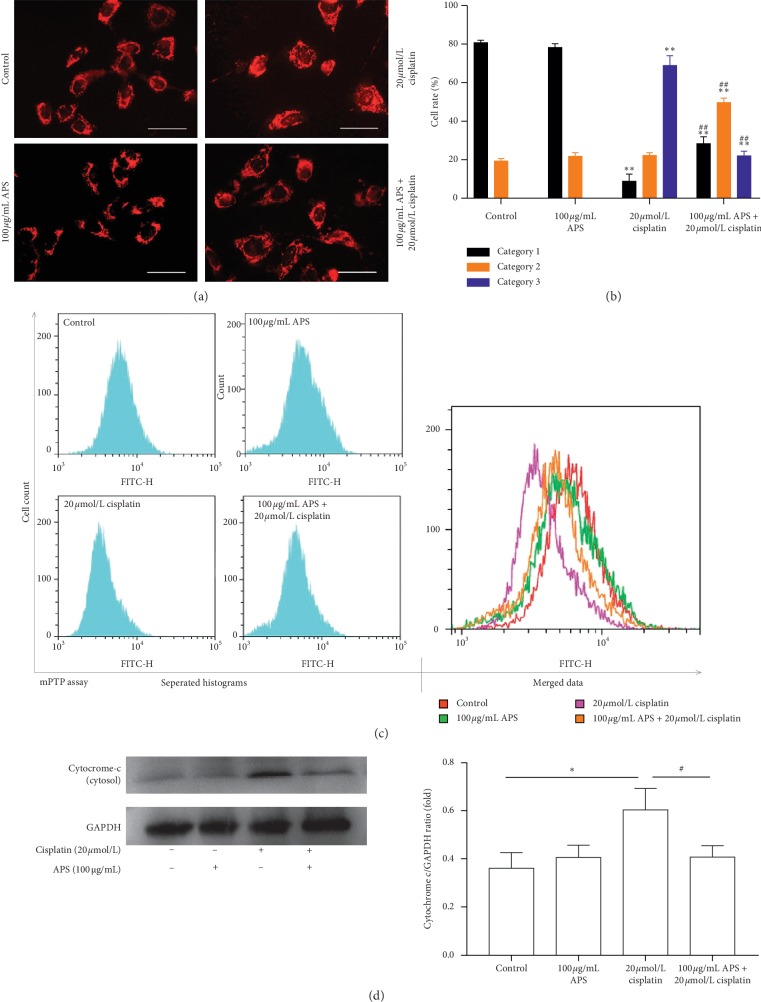
APS ameliorates the cisplatin-triggered mitochondrial dysfunction in HK-2 cells. (a) MitoTracker® Red CMXRos staining of HK-2 cells with different treatments (original magnification ×400; bar = 100 *μ*m). (b) Quantification of mitochondrial morphology as category 1 (elongated tubulin-like structure), category 2 (large round or short tubular), and category 3 (round or punctate) in 6 random fields. (c) Representative flow cytometry curves of the HK-2 cells stained by mPTP assay kit. (d) Western blot analysis of cytoplasmic cytochrome *c* level in HK-2 cells with different treatments. Graphs showing the results of quantitative analysis are shown on the right. Data are means ± SEM (*n* = 3). ^*∗*^*P* < 0.05, ^*∗∗*^*P* < 0.01 versus the control group; ^#^*P* < 0.05, ^##^*P* < 0.01 versus the cisplatin alone-treated group.

Mitochondrial-mediated apoptosis is considered as an important mechanism in cisplatin-induced cell death [29]. TEM was used to observe the mitochondrial morphology in mice renal. As shown in Figure 5(a), the mitochondria kept intact membrane and obvious cristae in the control group. After the treatment of cisplatin, the mitochondria of mice renal showed obvious vacuolation and swelling. However, APS pretreatment seems to partially reverse these damages induced by cisplatin. The quantification of mitochondrial vacuolation indicated that APS pretreatment significantly improved the integrity status of mitochondria (Figure 5(b)). To confirm these results, mitochondrial function was investigated in cisplatin-treated HK-2 cells in our study. To determine whether APS attenuated cisplatin-induced cytotoxicity in HK-2 cells through protecting mitochondria, the changes of mitochondrial morphology were detected by MitoTracker staining. As shown in Figure 6(a), in the control group, normal mitochondria mostly exhibited interconnected long tubular network (category 1). Mitochondria remained normal in APS-treated cells, whereas in cisplatin-treated cells, mitochondria became fragmented, displaying large round or short tubules (category 2) and round fragments (category 3). Interestingly, these cells that pretreated with APS exhibited a significantly lower rate of mitochondria in category 3 and a higher rate of category 1 than cells treated with cisplatin alone (Figure 6(b)).

The mitochondrial permeability was evaluated using an mPTP assay kit (flow cytometry). As shown in Figure 6(c), compared with the control group, mPTP remained normal in APS-treated cells. However, cisplatin caused significant weakening of mitochondrial fluorescence intensity, which indicated the opening of mPTP. APS pretreatment could significantly block the opening of mPTP, showing stronger mitochondrial fluorescence intensity than the cisplatin alone-treated group. When the mitochondria became permeable, apoptosis-inducing proteins in mitochondria such as cytochrome *c* would leak into the cytoplasm and activated the apoptotic pathway. We further determined the cytoplasmic cytochrome *c* level in HK-2 cells. As shown in Figure 6(d), cytoplasmic cytochrome *c* expression was significantly increased after cisplatin exposure. And the leakage of cytochrome *c* could be significantly reduced with APS pretreatment. Those results indicated that APS could potentially alleviate mitochondrial-mediated apoptosis through protecting mitochondria and reducing cytochrome *c* release.

## 4. Discussion

Nephrotoxicity is a disturbing complication of cisplatin chemotherapy, which lacks effective prevention [26]. Therefore, exploring a novel therapeutic strategy to prevent this dilemma is meaningful for cisplatin in cancer therapy. APS is a main bioactive component of Astragalus membranaceus, which has been reported to exhibit multiple biological activities including anti-inflammatory, antioxidant, antiaging, and mitochondria protection [15, 19, 30, 31]. Based on these properties of APS in many pathological processes, we explored its effect on cisplatin-induced AKI in this study. We demonstrated that APS could alleviate cisplatin-induced renal tubular epithelial cells injury both in vivo and in vitro. APS appeared to inhibit cisplatin-induced oxidative damage and alleviate mitochondrial-mediated apoptosis through protecting mitochondria and reducing cytochrome *c* leakage.

Tubular epithelial cells in the kidney are anticipated to be the main targets of cisplatin toxicity [9]. In our study, cisplatin (30 mg/kg) administration in mice caused a marked kidney injury, showing elevation of kidney injury markers in serum and tissue, accompanying with obvious histopathological damage in renal tubules. Interestingly, these changes of the kidney could be attenuated by APS pretreatment in mice. KIM-1 is recognized as a sensitive biomarker for early detection of cisplatin-induced AKI [32]. Here, KIM-1 level in serum and renal tissue was markedly elevated in mice treated with cisplatin. However, it was significantly reduced when mice were pretreated with APS before cisplatin injection. Similarly, in the HK-2 cell study, APS could protect the viability of HK-2 cells upon cisplatin exposure. Those in vivo and in vitro data indicated that APS possessed a protective effect on cisplatin-induced kidney toxicity.

Cisplatin-induced oxidative damage has been demonstrated to be important for the development of kidney injury, resulting from both excessive ROS accumulation and antioxidant enzymes suppression, including T-SOD and GSH-Px. ROS is found to promote inflammatory response and cell apoptosis [33], whereas T-SOD and GSH-Px are endogenous molecules that can scavenge ROS and protect cells from oxidative damage [34]. Our results showed that cisplatin markedly induced oxidative damage, characterized by a significant accumulation of oxidative stress marker 3-NT and inhibition of antioxidant enzyme activities, including T-SOD and GSH-Px in mice kidney tissues. In addition, cisplatin markedly increased intracellular ROS accumulation in HK-2 cells. Several studies have reported that ROS-specific inhibitor effectively alleviated cisplatin-induced kidney toxicity by reducing ROS accumulation [35]. APS has been reported to be a natural antioxidant in various pathological states [14, 18, 36, 37]. As anticipated, in our study, APS also could mitigate cisplatin-induced oxidative damage by reducing ROS generation and recovering the activities of T-SOD and GSH-Px. It has been reported that cisplatin-induced oxidative stress primarily originates from mitochondria [6]. Similarly, we observed obvious mitochondrial ROS generation in cisplatin-treated HK-2 cells, while these changes could be ameliorated when cells were pretreated with APS, which further identified the antioxidative property of APS in cisplatin-induced oxidative damage.

Mitochondria are abundant in renal tubular epithelial cells and play an important role in maintaining normal cell physiological function [9]. Mitochondrial dysfunction plays a crucial role in cisplatin-induced renal injury, including morphologic changes of mitochondria, mitochondrial membrane permeabilization, and leakage of apoptosis-inducing proteins into the cytoplasm [7, 28, 38]. In our study, we found that cisplatin induced obvious mitochondrial vacuolation and swelling in mice renal; interestingly, APS pretreatment seems to partially reverse these damages induced by cisplatin, indicating a protective effect on integrity status of mitochondria. In HK-2 cells, cisplatin also significantly induced fragmentation of mitochondria and opening of mPTP. Interestingly, the mitochondrial dysfunction could also be ameliorated by APS through maintaining the normal morphology of mitochondria and blocking the opening of mPTP. In addition, mitochondria are initial regulators of cell apoptosis and contain apoptogenic proteins, such as cytochrome *c* [39, 40]. Our results demonstrated that cisplatin significantly promoted leakage of cytochrome *c* into the cytoplasm and induced significant cell apoptosis in both mice kidney tissue and HK-2 cells, displaying increased number of TUNEL-positive cells. Interestingly, APS significantly reduced cisplatin-induced cytochrome *c* leakage and cell apoptosis. Here, these results indicated that the protective effect of APS on mitochondria was one possible mechanism through which APS ameliorated cisplatin-induced apoptosis in mice kidney and HK-2 cells.

In conclusion, our study indicates that APS pretreatment could attenuate cisplatin-induced AKI through inhibiting oxidative damage, protecting mitochondria, and ameliorating mitochondrial-mediated apoptosis. APS has been approved in the clinic in China for the adjuvant treatment of patients with cancer through intravenous injection, and it could be meaningful to confirm its protective effects on cisplatin-induced AKI in patients.

## Data Availability

The data that are used to support the findings of this study are available from the corresponding author upon reasonable request.
